# Effect of antimicrobial nanocomposites on *Vibrio cholerae* lifestyles: Pellicle biofilm, planktonic and surface-attached biofilm

**DOI:** 10.1371/journal.pone.0217869

**Published:** 2019-06-12

**Authors:** Anaid Meza-Villezcas, Ana L. Gallego-Hernández, Fitnat H. Yildiz, Oscar E. Jaime-Acuña, Oscar Raymond-Herrera, Alejandro Huerta-Saquero

**Affiliations:** 1 Centro de Investigación Científica y de Educación Superior de Ensenada, Ensenada, Baja California, México; 2 Centro de Nanociencias y Nanotecnología, Universidad Nacional Autónoma de México, Ensenada, Baja California, México; 3 Microbiology and Environmental Toxicology Department, University of California Santa Cruz, Santa Cruz, California, United States of America; VIT University, INDIA

## Abstract

*Vibrio cholerae* is an important human pathogen causing intestinal disease with a high incidence in developing countries. *V*. *cholerae* can switch between planktonic and biofilm lifestyles. Biofilm formation is determinant for transmission, virulence and antibiotic resistance. Due to the enhanced antibiotic resistance observed by bacterial pathogens, antimicrobial nanomaterials have been used to combat infections by stopping bacterial growth and preventing biofilm formation. In this study, the effect of the nanocomposites zeolite-embedded silver (Ag), copper (Cu), or zinc (Zn) nanoparticles (NPs) was evaluated in *V*. *cholerae* planktonic cells, and in two biofilm states: pellicle biofilm (PB), formed between air-liquid interphase, and surface-attached biofilm (SB), formed at solid-liquid interfaces. Each nanocomposite type had a distinctive antimicrobial effect altering each *V*. *cholerae* lifestyles differently. The ZEO-AgNPs nanocomposite inhibited PB formation at 4 μg/ml, and prevented SB formation and eliminated planktonic cells at 8 μg/ml. In contrast, the nanocomposites ZEO-CuNPs and ZEO-ZnNPs affect *V*. *cholerae* viability but did not completely avoid bacterial growth. At transcriptional level, depending on the nanoparticles and biofilm type, nanocomposites modified the relative expression of the *vpsL*, *rbmA* and *bap1*, genes involved in biofilm formation. Furthermore, the relative abundance of the outer membrane proteins OmpT, OmpU, OmpA and OmpW also differs among treatments in PB and SB. This work provides a basis for further study of the nanomaterials effect at structural, genetic and proteomic levels to understand the response mechanisms of *V*. *cholerae* against metallic nanoparticles.

## Introduction

*Vibrio cholerae* pathogenic strains are the etiologic agent of cholera, an acute watery diarrheal disease that occurs in 3–5 millions of persons annually, with 100,000 to 120,000 lethal cases [1,2]. *V*. *cholerae* can switch between planktonic and biofilm lifestyles. Biofilms are microbial communities, composed of microorganisms and extracellular matrix [3]. Biofilm formation enhances growth, survival, and persistence of *Vibrio* species in the aquatic ecosystem, and also increase its antibiotic resistance [4,5]. In the human host, biofilms play a role in the disease process, with a higher infectivity than planktonic cells [6,7].

Looking for alternative treatments, evaluation of metallic nanoparticles against *V*. *cholerae* has been conducted with favorable results. *V*. *cholerae*, and other *Vibrio* species such as *V*. *parahaemolyticus*, *V*. *vulnificus*, *V*. *fischeri*, *V*. *harveyi* and *V*. *alginolyticus* have been shown to be susceptible to the effect of nanomaterials, such as silver, copper oxide, zinc oxide, boron, titanium dioxide and silver-doped zeolites [7–18]. Previous studies revealed an antibacterial effect of zinc-oxide nanoparticles (ZnONP) and silver nanoparticles (AgNPs) against *V*. *cholerae*, depends on its lifestyle (planktonic or biofilm); serogroups (O1 or O139) or biotypes (Classical or El Tor); and this effect is also dependent on the nanomaterial composition and concentration [7,10,19,20]. Structural analysis of planktonic cells treated with ZnONPs and AgNPs showed alteration in the bacterial size and shape; in particular, ZnONPs increase the cell membrane fluidity and the reactive oxygen species generation and concomitantly, DNA damage [10,20]. It was shown that AgNPs were able to penetrate approximately 40 μm in a thick biofilm after 1 h of exposure, suggesting that biofilm resistance to AgNPs could be at least partially due to retarded silver ion/particle diffusion. A similar effect of the bacteria cell protection in biofilms has been proposed for the salts of copper, lead, zinc and silver ions in *P*. *aeruginosa* [21,22]. From the little information that we have about pathogenic bacteria molecular response to metallic nanoparticles, most of the research has been focus on planktonic cells, which might not be the only lifestyle of some microorganism in the natural environment.

The outer membrane proteins (OMPs) also have an impact on bacterial resistance to nanoparticles. In *Escherichia coli*, mutants deficient in OmpF and OmpC porins showed an increase in resistance to AgNPs [23]. In *V*. *cholerae*, in the presence of sub-lethal concentrations of AgNPs and ZnONPs, changes on the relative expression of outer membrane proteins such as OmpT and OmpU were reported [7]. In the present study, the antimicrobial properties of CEOBACTER (AgNP-doped zeolites, ZEO-AgNPs) [24,25], and the new nanocomposites ZEO-CuNPs, and ZEO-ZnNPs (copper and zinc nanoparticles-doped zeolites, respectively) were evaluated on three different lifestyles of *Vibrio cholerae* O1 biotype El Tor A1552: pellicle biofilm (PB), planktonic cells, and surface-attached biofilm (SB) [26]. We analyzed changes on gene expression of *vpsR*, *vpsL*, *rbmA* and *bap1*, genes involved in biofilm formation. At protein level, the relative abundance of the outer membrane proteins among treatments was evaluated in the PB and SB. Taking together, the differential response of PB and SB triggered by antimicrobial nanomaterials allows us to get insight into the *V*. *cholerae* survival mechanisms.

## Materials and methods

### Synthesis and characterization of the silver, copper and zinc zeolite nanocomposites

Silver, copper and zinc zeolite nanocomposites (ZEO-AgNPs, ZEO-CuNPs, and ZEO-ZnNPs) were synthesized following the methodology described in the patent application MX/a/2012/013218 and in previous work [24,25]. ZEO-AgNPs, ZEO-CuNPs, and ZEO-ZnNPs were characterized by X-ray diffraction (XRD, Philips X’Pert diffractometer-CuKα radiation), by scanning electron microscopy (SEM, JEOL JSM-5300 microscope) and by transmission electron microscopy (TEM, JEOL JEM-2010 with accelerating voltage of 200 kV). The nanocomposites were visualized before and after 48h of treatment in Lysogeny Broth (LB) culture by TEM to evaluate the possible effect of the treatment on their structure. A global chemical analysis was performed by inductively coupled plasma-atomic emission spectroscopy (ICP-AES, Variant Liberty 110 Spectrometer). The electronic state of the Ag, Cu, or Zn atoms and the oxidation state of the nanoparticles was study with X-ray photoelectron spectroscopy (XPS) in a SPECS system equipped with a PHOIBOS WAL electron energy analyser using a monochromatic Al anode with an accuracy of the binding energy (BE) values is ± 0.1 eV. The Z-potential of the ZEO-AgNPs, ZEO-CuNPs, and ZEO-ZnNPs were analysed before and after 48h of treatment in Lysogeny Broth (LB) culture media (ZETASIZER, Malvern).

### Bacterial growth and treatments conditions

*Vibrio cholerae* O1 El Tor (strain A1552 rugose variant) was used in these studies. The variant rugose is a wild-type *Vibrio cholerae* O1 biotype El Tor A1552 derivate with improved biofilm production [26]. *V*. *cholerae* was cultured at 30 °C in Lysogeny Broth (LB) broth or agar media (broth: 1% tryptone, 1% sodium chloride, 0.5% yeast extract; agar plates: 2% bacteriological agar added, adjusted to pH 7.5). *V*. *cholerae* colonies were cultured overnight at 30 °C and 200 rpm in 24-well flat bottom plates. Bacterial susceptibility test was performed with an initial bacterial inoculum of 1x10^4^ CFU prepared from the overnight LB culture. *V*. *cholerae* was grown in the absence and presence of different concentrations of zeolite (ZEO) as a control, ZEO-AgNPs (1.0, 2.0, 4.0 and 8.0 μg/ml), ZEO-CuNPs and ZEO-ZnNPs (160.0, 320.0 and 420.0 μg/ml), for 48 h at 30 °C, in static condition to promote pellicle biofilm (PB) and surface-attached biofilm (SB) formation. The equivalent of the highest ZEO concentration of nanocomposites (28 mg) was used for the ZEO as our control. At 48 h post-incubation, the PB was formed as a thin floating layer between air-liquid interphase; the planktonic cells were found in suspension in the liquid interface; and SB was found at the bottom of culture-well. Experiments were done in triplicates with at least 3 biological replicates.

### Bacterial viability test

*V*. *cholerae* viability was checked by calculating CFU/ml. Briefly, the PB was removed carefully from the air-liquid interface using bacteriological loops to avoid disruption, and resuspended in 1 ml PBS (pH 7.4). For planktonic lifestyle, 1 ml of culture was transferred to individual tubes, centrifuged and resuspended in 1 ml of PBS. SB sample was obtained by resuspending cells attached to the bottom of the well with 1 ml PBS (pH 7.4). For PB, planktonic cells, and SB the CFU/ml were calculated by serial dilution method with the following modification: biofilms and microaggregates formed were dispersed in each dilution by vortexing for 30s with 3-mm glass beads [26]. To demonstrate inertness of sodium-mordenite matrix (ZEO) on bacterial growth, ZEO without metallic nanoparticles were tested following the procedure described above. The minimal bactericidal concentration (MBC) was defined as the minimum nanoparticle concentration to kill 99.9% of planktonic cells, whereas the minimal biofilm eradication concentration (MBEC) was defined as the minimum concentration of nanoparticles required to prevent PB and SB formation [27,28].

### Ultrastructural analysis by scanning electron microscopy

PB, planktonic, and SB treated with 1 μg/ml of ZEO-AgNPs and 420 μg/ml ZEO-CuNPs and ZEO-ZnNPs were fixed on a round-coverslip (12 mm) with 2.5% glutaraldehyde for 1 h. Next, the samples were washed and gradually dehydrated with 10%, 25%, 50%, 75%, 90% and 100% ethyl alcohol (Sigma Aldrich), at room temperature. The samples were critically point dried (model: Balzers Union 342/11 120B), sputtered with ~20 nm of gold (model: Technics Hummer VI). The samples were visualized with a FEI Quanta 3D Dualbeam SEM operating at 5 kV and 6.7 pA at the SEM facility at University of California Santa Cruz.

### RNA isolation and quantitative RT-PCR

Total RNA was isolated from PB and SB non-treated and treated with nanocomposites at 1 μg/ml of AgNPs and 420 μg/ml CuNPs and ZnNPs using TRizol reagent (Invitrogen) after 48 hours of growth. The RNA extraction was purified by TURBO DNA-free TURBO DNase kit (Invitrogen) and RNeasy MinElute kit (QIAGEN). The pooled RNA (100 ng) was converted to one-strand cDNA using SuperScript reverse transcriptase. Then, quantitative real-time PCR (RT-qPCR) was performed in PikoReal 96 Real-Time PCR System (Thermo Fisher) using specific primers to *vpsR*, *vpsL*, *rbmA*, and *bap1* genes; as well as *dnaE* gene as a control (Table 1), by means of SsoAdvanced Universal SYBR Green Supermix (2x) (BioRad). Data analysis was done according to the Livak method (fold difference = 2- ΔΔCt) [29].

**Table 1:** Oligonucleotides used in this study.

Gene ID	Name	Forward primer	Reverse primer
VC0665	*vpsR*	CTCTTGTTGTGGTGGGAGGT	GGCCCAGTCTCGACAAATAA
VC0934	*vpsL*	CCTTGCTAGGGGTGCTTTTT	TAGGGTCAAGCTAGCGATGG
VC0928	*rbmA*	TGGCAAGTAACGGTGGATAC	GCTTTGGCTGGGAAGTAGAT
VC1888	*bap1*	GGCGAGTCAACAACCTATCT	CCAGTCGGTGTAGCCATAAA
VC2245	*dnaE*	CCCGGTTACTCAGTTCGATAAG	AGCCCAGTCAATAATGGTAAGG

### Outer membrane proteins isolation

Total proteins were isolated from PB and SB non-treated and treated with 1 μg/ml of ZEO-AgNPs, and 420 μg/ml ZEO-CuNPs and ZEO-ZnNPs. PB and SB were resuspended in 1 ml of PBS pH 7.4, then centrifuged 20 min at 12,000 x g, at 4 °C. The cells were resuspended in 2.5 ml of 10 mM Tris pH 8–0.5 mM PMSF (Sigma Aldrich). The cell lysis was performed by sonication (Ultrasonic cell crusher YM-1000Y). Insoluble material was pelleted at 8,000 xg 20 min at 4 °C. Then, the membrane fraction was separated from the supernatant at 20,000 rpm 60 min at 4 °C. The membrane fraction was treated with 1 ml of 1.67% sodium N-lauroylsarcinosinate for 20 min at room temperature, followed by centrifugation 90 min at 20,000 rpm and 4 °C. The pellet contained the outer membrane proteins was resuspended in 30 μl of 10 mM Tris pH 8–0.5 mM PMSF. Total protein was quantified by Bradford reagent (Sigma Aldrich). Electrophoresis was carried out loading 1 μg of protein for each treatment in 15% SDS-PAGE and stained with SYPRO Ruby (Thermo Fisher Scientific). Densitometric analysis was performed using ImageJ software defining a selection as Region of Interest (ROI) and measuring the pixel intensities of each protein for treatment (https://imagej.nih.gov). Three independent OMPs purification experiments were performed (n = 3). For the statistical analysis, the abundance of each OMP was normalized with ZEO treatment considered as 1.

### Protein isolation and immunoblotting

The molecular weight of OmpU and OmpT were verified by immunobloting. *V*. *cholerae* total protein was isolated from cultures by standard procedures, and resuspended in 500 μl of 2% SDS (sodium dodecyl sulfate) ionic detergent, heated to 95 °C for 10 min, and centrifuged 2 min at 12,000 xg. The supernatant phase containing the total protein extract was quantified by Pierce BCA Protein Assay Kit (Thermo Fisher Scientific). SDS-PAGE was carried out loading 30 μg of the protein of each treatment per lane and then transferred by Semi-Dry Trans-Blot (BioRad) onto a polyvinylidene difluoride membrane (PVDF). Immunodetection was performed using the rabbit anti-OmpT (1:1,000) and goat anti-OmpU (1:1,000), followed by the incubation with the secondary antibodies anti-rabbit IgG-HRP (1:2,500) and anti-goat IgG-HRP (1:2,500), respectively. The immunoblot was visualized using the HRP SuperSignal West Pico Chemiluminescent Substrate (Thermo Fisher Scientific).

### Statistical analysis

Data were analysed using one-way ANOVA by ranks test followed by post hoc Dunnett´s multiple comparisons versus control group. Differences were considered significant at p-values of ≤ 0.05. For all statistical analyses SigmaPlot (Systat Software, San Jose, CA) was used.

## Results

### Nanocomposites characterization

Nanocomposites containing metallic nanoparticles of silver, copper or zinc embedded on the surface of mordenite-type zeolite, were synthesized by a one-pot, solvent-free and organic template-free variant of the sol-gel process [24]. The resulting nanocomposites, ZEO-AgNPs, ZEO-CuNPs, and ZEO-ZnNPs, were insoluble powders as observed in scanning electron microscopy (SEM) images (S1 Fig), consisting of spheroidal-shape grains with 25 μm of average diameter, formed by packing of needle-shaped mordenite crystals. X-ray diffraction (XRD) patterns exhibit good defined single phase mordenite-type zeolite structure with high crystallinity, where no reflections of the metallic silver, copper, or zinc were present as evidence of the absence of bulk-like particles [24,25,30]. To demonstrate the presence of the nanoparticles and to evaluate their size, AgNPs, CuNPs and ZnNPs were visualized by transmission electron microscopy (TEM) before and after 48h of culture media treatment, and no changes due to media and incubation time were detected (S2 Fig). After the synthesis, the average size of the nanoparticles was 3–15 nm for all samples. The metal concentration of Ag, Cu, and Zn was quantified by inductively coupled plasma-atomic emission spectroscopy (ICP-AES), and showed a concentration of 15 ppm, 12.6 ppm, and 18 ppm, respectively. The Zeta-potential analysis showed the good quality of the nanocomposites before and after treatment, with an average value of -15.50 mV before treatment and -18.15mV after 48 hours of treatment (S1 Table). Due to the synthesis method, the advantage of these nanoparticles is that they do not detach from the crystalline zeolite structure, and more importantly, the crystalline structure allows liquid permeation and ions release, which carry out the antimicrobial effect [25]. The nanoparticles immobilization hinders their oxidation, degradation and agglomeration, favoring long-term antimicrobial activity, as well as preventing detachment. To know the oxidation state of the metallic NPs and to confirm the bonding of NPs on the zeolite surface, X-ray photoelectron spectroscopy (XPS) measurements were performed (S3 Fig). The results for Ag-3d, Cu-2p and Zn-2p XPS spectra were collected for ZEO-AgNPs, ZEO-CuNPs and ZEO-ZnNPs, respectively, and the NIST X-ray Photoelectron Spectroscopy Database was used for analysis (https://srdata.nist.gov/xps/Default.aspx). In agreement with the XRD and TEM results, the NPs for all nanocomposites can be considered with a core-shell type structure where the metallic cores of Ag, Zn, or Cu are covered by AgO for ZEO-AgNPs, ZnO for ZEO-ZnNPs, or complex shells constituted by oxide species as Cu(OH)_2_, Cu_2_O and CuO for ZEO-CuNPs. On the other hand, the presence of spectral lines characteristic of Ag^+^ for ZEO-AgNPs, of the compounds Al_2_CuO_4_ for ZEO-CuNPs, and Al_2_ZnO_4_ and ZnAl_2_O_4_ for ZEO-ZnNPs are taken as evidence that, for all nanocomposites, the NPs are strongly bonded to the surface of the zeolite. In particular, the high presence of the spectral line of Zn-2p1/2 at 1044 eV, associated to ZnAl_2_O_4_ compound, is indicative that ZnNPs grow deeper in the zeolite matrix with lower surface exposed to environment, in correspondence with the TEM analysis (S2 Fig).

### The nanocomposites showed a specific antimicrobial effect and response to the nanocomposites depends on lifestyles of *V*. *cholerae*

The antimicrobial properties of each metallic nanoparticles embedded in a crystalline zeolite structure, ZEO-AgNPs, ZEO-CuNPs, and ZEO-ZnNPs were evaluated against *V*. *cholerae* rugose variant, which promotes biofilm production and forms PB in a shorter period of time compare to the wild-type (72 h) [26]. *V*. *cholerae* rugose variants has been isolated during cholera epidemics in Latin America, it can cause a typical cholera diarrheal illness when orally administered and has been associated with increased survival in chlorinated water when compared with the wild-type strain (smooth phenotype) [31,32].

Static cultures in 24-well plates were grown to determine the minimal biofilm eradication concentration (MBEC) in pellicle biofilm (PB) and surface-attached biofilm (SB), as well as the minimal bactericidal concentration (MBC) in planktonic cells. Under this growth condition and after 48-hours of incubation, the cellular culture was composed of the PB, located in the air-liquid interface; planktonic cells, present in the liquid media; and SB aggregates, attached at the bottom surface of the well.

**Fig 1:**
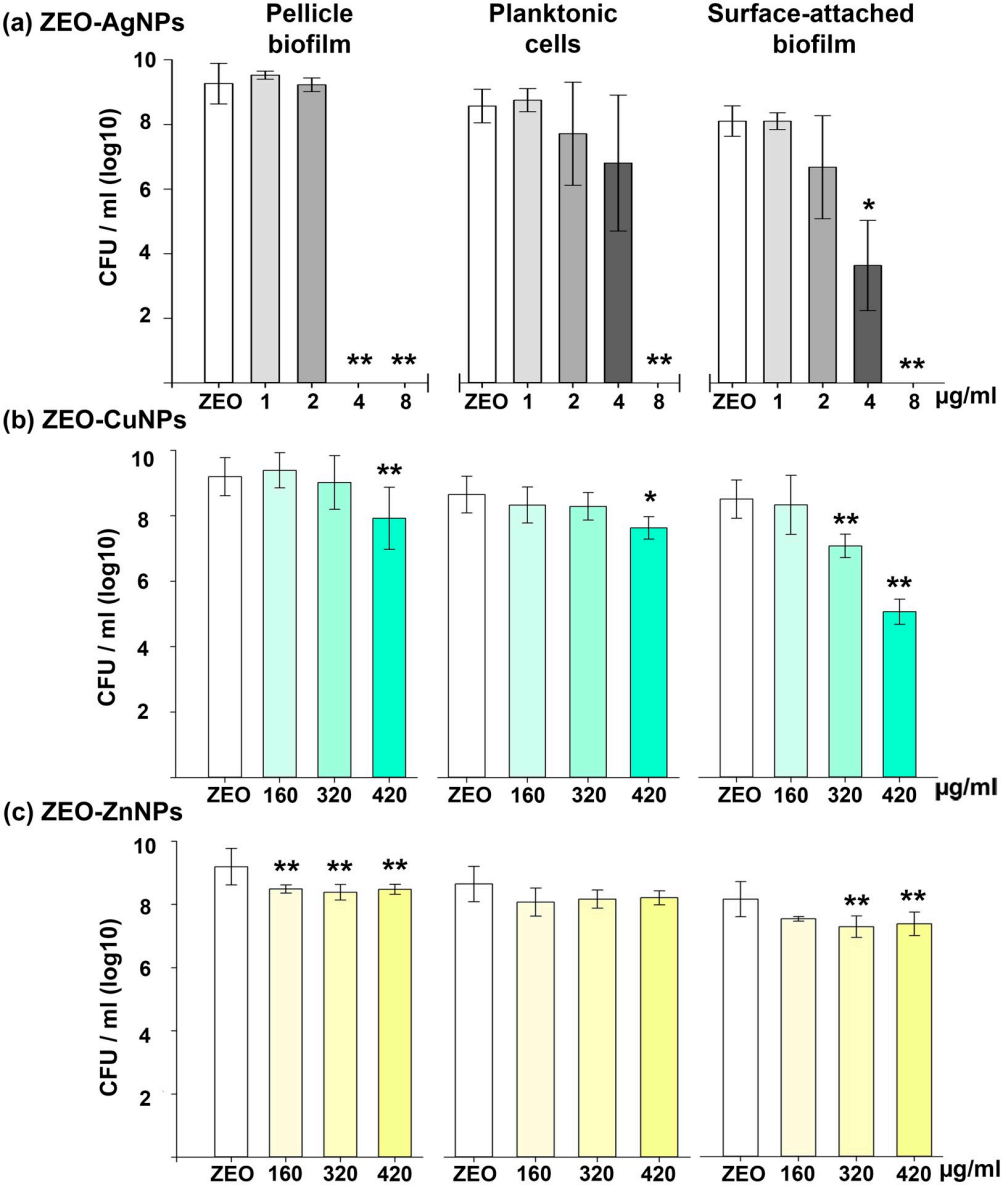
Antimicrobial effect of the nanocomposites on pellicle biofilm (PB), planktonic cells, and surface-attached biofilm (SB) of *V*. *cholerae*. Samples were exposed to different concentrations of (a) ZEO-AgNPs, (b) ZEO-CuNPs, and (c) ZEO-ZnNPs; (ZEO was used as control). Colony-forming unit per ml (CFU/ml) was determined by plate count. Treatments was performed by triplicate, values shown from a representative experiment are means (SD) of each treatment. Significant changes are marked with asterisk: * p-value < 0.05, ** p-value <0.01.

ZEO-AgNPs MBEC for PB was detected at 4 μg/ml, whereas the MBC and the MBEC for planktonic and SB increase to 8 μg/ml, respectively (Fig 1A). Our results are consistent with previous results, ZEO-AgNPs MBC has been determined for *E*. *coli* and other Gram-negative bacteria with concentrations ranging from 3 to 10 μg/ml [24,33].

We were not able to determine MBEC and MBC for ZEO-CuNPs and ZEO-ZnNPs (Fig 1B and 1C). The highest concentration tested was 420 μg/ml and higher concentrations were not analyzed due to saturation of the nanocomposite solutions thwarts manipulation. Nevertheless, in PB and planktonic cells, ZEO-CuNPs reduced significantly the cell-forming units (CFU) number at 420 μg/ml; meanwhile, for SB a significant decrease in viability was observed after 320 μg/ml (Fig 1B). With ZEO-ZnNPs treatment, PB and SB showed a significant CFU reduction at 160 μg/ml and 320 μg/ml, respectively, and planktonic cells viability does not seem to present a significant change (Fig 1C).

The results showed that the three nanocomposites tested have an impact in cell survival in *V*. *cholerae* PB and SB, and the overall antimicrobial effect of each nanocomposite type was different and dependent on *V*. *cholerae* lifestyle.

### The nanocomposites do not modify the pellicle biofilm, planktonic cell and surface-attached biofilm structures, but they do modify the substrate attachment

**Fig 2:**
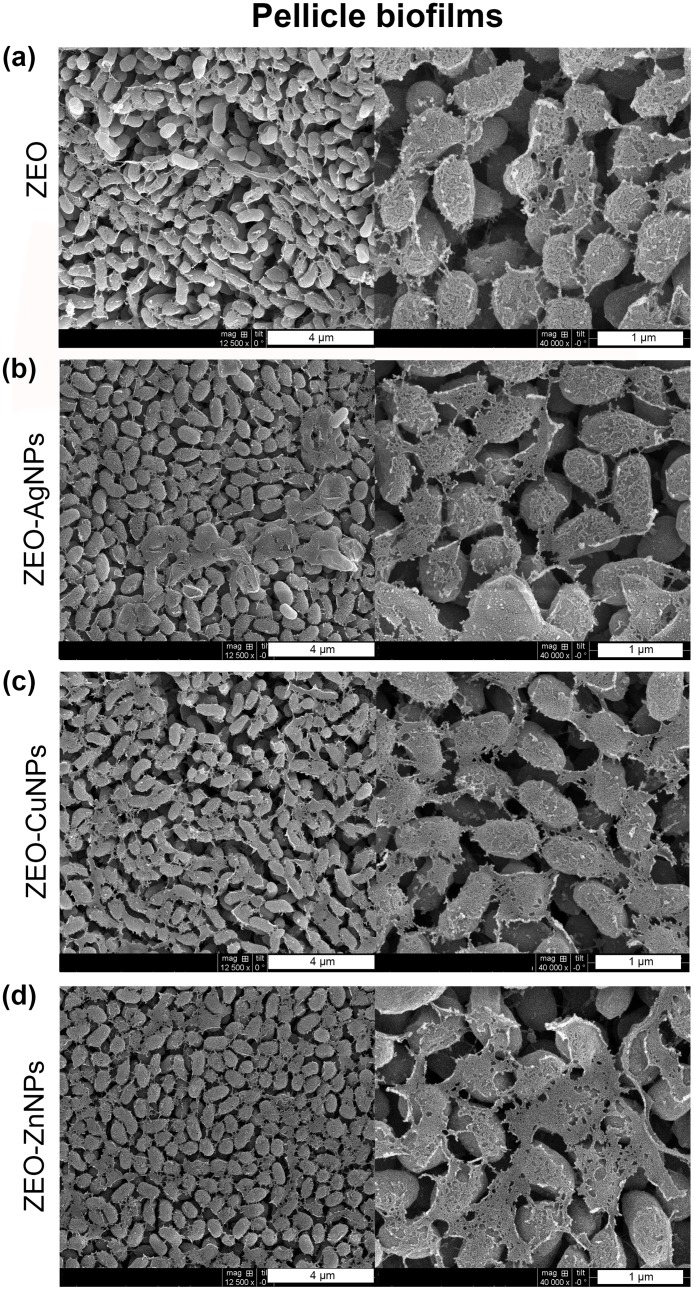
Structural analysis of pellicle biofilms exposed to nanocomposites. Scanning electron microscopy (SEM) of *V*. *cholerae* treated with (a) ZEO as a control, (b) ZEO-AgNPs (1 μg/ml), (c) ZEO-CuNPs (420 μg/ml) and, (d) ZEO-ZnNPs (420 μg/ml. Representative images of n = 3 biological replicates are shown.

**Fig 3:**
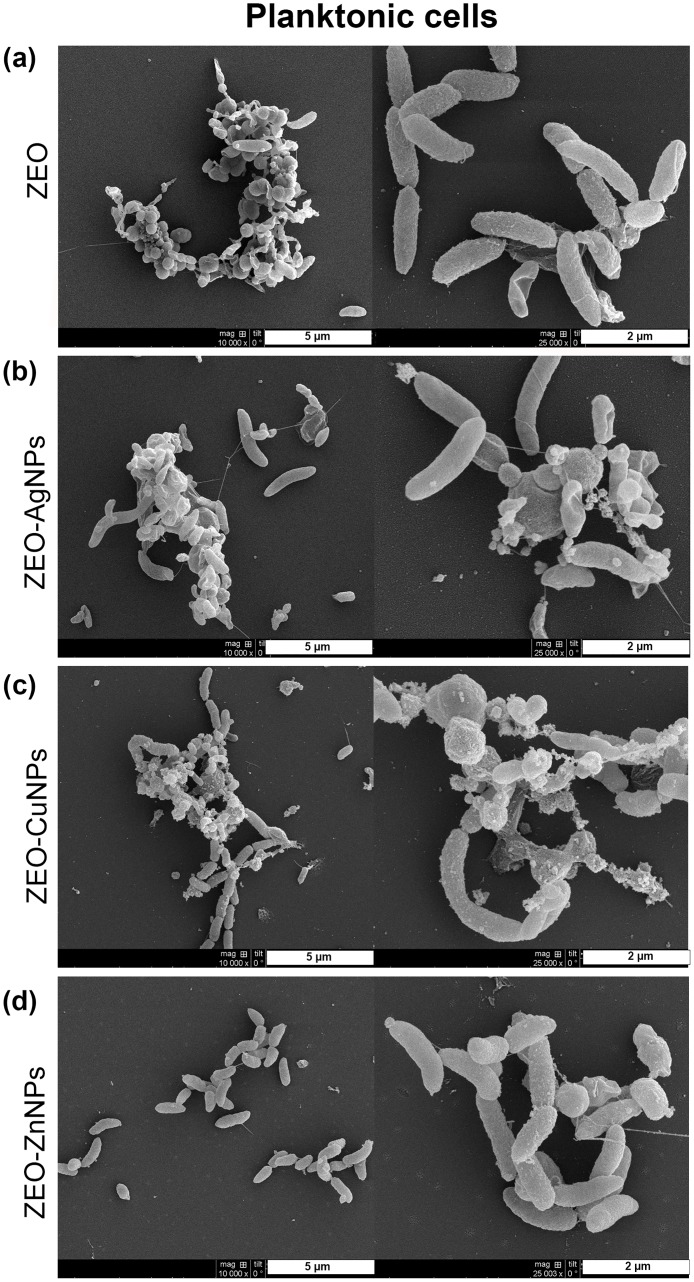
Structural analysis of planktonic cells treated with nanocomposites. Scanning electron microscopy (SEM) images of *V*. *cholerae* treated with (a) ZEO as a control, (b) ZEO-AgNPs (1 μg/ml), (c) ZEO-CuNPs (420 μg/ml) and, (d) ZEO-ZnNPs (420 μg/ml). Representative images of n = 3 biological replicates are shown.

**Fig 4:**
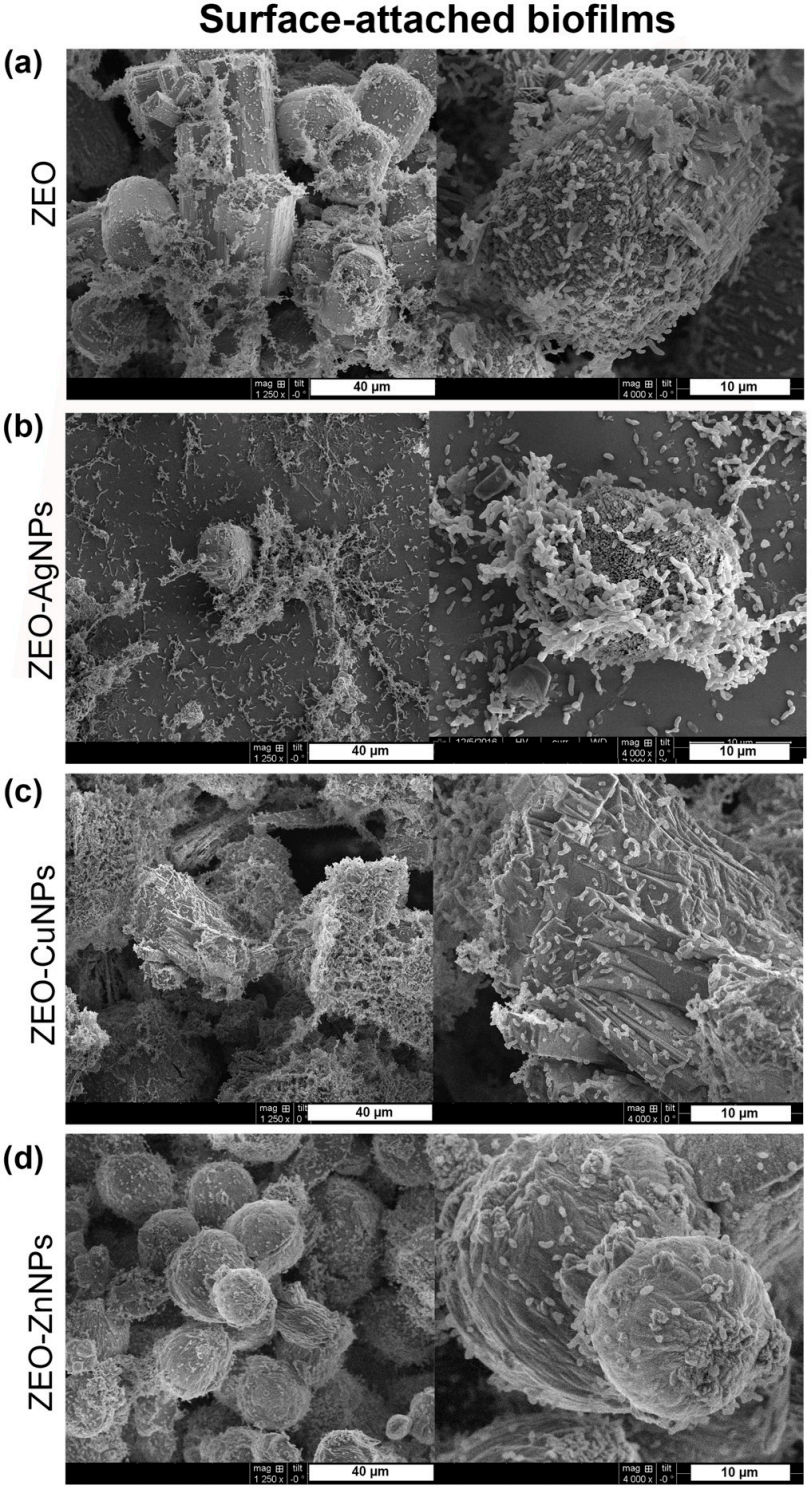
Structural analysis of surface-attached biofilms treated with nanocomposites. Scanning electron microscopy (SEM) was performed of *V*. *cholerae* treated with (a) ZEO as a control, (b) ZEO-AgNPs (1 μg/ml), (c) ZEO-CuNPs (420 μg/ml) and, (d) ZEO-ZnNPs (420 μg/ml). Representative images of n = 3 biological replicates.

Next, we sought to determine the effect of ZEO-AgNPs, ZEO-CuNPs, and ZEO-ZnNPs on the structure of the *V*. *cholerae* PB, planktonic cells, and SB by scanning electron microscopy (SEM). For this purpose, *V*. *cholerae* cultures were grown for 48 h with sublethal concentrations of the nanocomposites. As a control, *V*. *cholerae* was incubated with and without the ZEO (zeolite matrix without metallic nanoparticles) to demonstrate that ZEO does not alter cellular morphology (S4 Fig). After treatments with 1 μg/ml of ZEO-AgNPs, and 420 μg/ml of ZEO-CuNPs or ZEO-ZnNPs, PB did not show structural modifications in comparison with the control (Fig 2). Planktonic cells treated with ZEO-AgNPs, ZEO-CuNPs, and ZEO-ZnNPs, as well as in the control condition, cells present drastic changes in morphology and formed micro colonies in all the conditions tested (Fig 3). However, structural modifications found in the cells might be due to the incubation time and not to the nanocomposite treatments or fixation technique, since 24 h of incubation of the control’s cultures provoked such structural changes, whereas shorter periods (6 and 12 h), maintained cellular morphology characteristic of *V*. *cholerae* (S5 Fig). In SB, the cells were aggregated and attached in a random manner to the zeolite crystal surfaces (Fig 4A). However, ZEO-CuNPs, and ZEO-ZnNPs treatments altered SB formation on zeolite crystals and surrounded surface (Fig 4B–4D). These observations agree with the data from the CFU/ml quantification, showing a reduction in the CFU/ml number in the presence of the ZEO-CuNPs, and ZEO-ZnNPs nanocomposites.

### The genes involved in the *Vibrio* polysaccharide and matrix protein biosynthesis are differentially expressed depending on nanocomposite treatments and *V*. *cholerae* lifestyles

**Fig 5:**
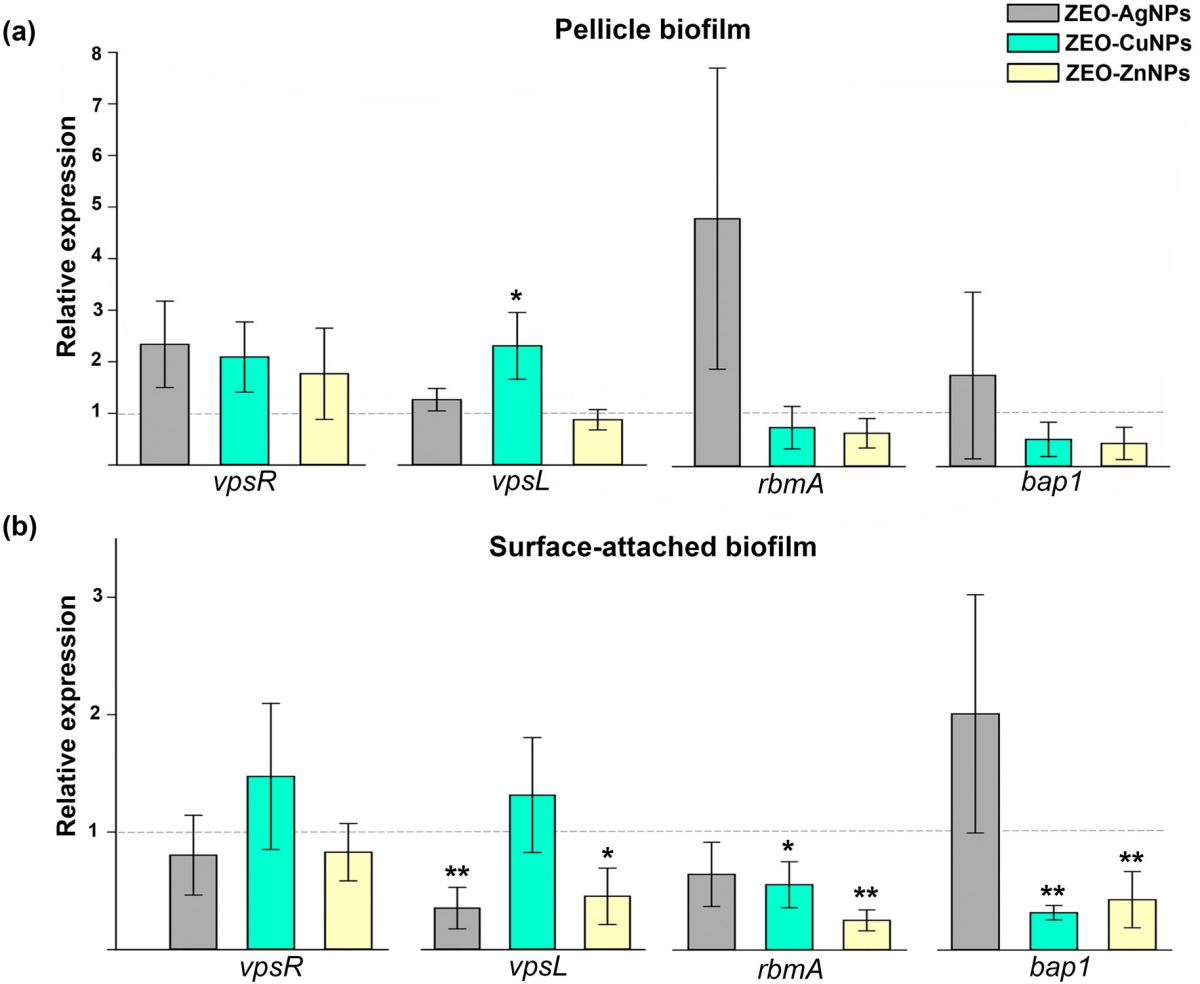
Relative expression of genes involved in biofilm formation after nanocomposites treatment. (a) Pellicle biofilm (PB) and (b) surface-attached biofilm (SB), treated at 1 μg/ml of ZEO-AgNP (gray), 420 μg/ml of ZEO-CuNPs (green) and ZEO-ZnNPs (yellow). A quantitative RT-PCR assay and Livak method analysis were used. The means (SD) values are shown (n = 3). The expression of each gene with ZEO were considered as 1 (horizontal line); ZEO as the zeolite matrix without metallic nanoparticles (control). For PB and SB, *dnaE* expression was used as internal control gene. Significant changes are marked with asterisk: * p-value < 0.05, ** p-value <0.01.

The differences we found on *V*. *cholerae* viability as well as in the biofilm formation in the presence of different nanocomposites, led us to hypothesize whether *V*. *cholerae* modifies the expression of biofilm-related genes after the treatment with metallic nanoparticles. In that sense, the relative expression of genes involved in *Vibrio* polysaccharide (VPS) and matrix protein biosynthesis were quantified by real-time RT-qPCR in PB and SB exposed to sub-lethal concentrations of ZEO-AgNPs, ZEO-CuNPs, and ZEO-ZnNPs. In PB, an increase on *vpsL* transcriptional levels, a gene involved in VPS production, was observed in ZEO-CuNPs nanocomposite treatment. Moreover, no significant differences in the relative expression of the biofilm master regulator *vpsR*, as well as the matrix proteins encoded genes, *rbmA* and *bap1*, was observed on all the treatments analyzed (Fig 5A). In SB, a significant decrease in the *vpsL* expression was found in the ZEO-AgNPs and ZEO-ZnNPs nanocomposites treatments, while no significant difference was found in ZEO-CuNPs treatment (Fig 5B). For the matrix genes *rbmA* and *bap1*, expression showed a significant decrease in both ZEO-CuNPs and ZEO-ZnNPs treatments, while no significant difference was found in the ZEO-AgNPs treatment (Fig 5B). Interestingly, the data obtained from the transcriptional analysis showed very different regulation between PB and SB aggregates; moreover, differences in expression are also dependent on the metallic NPs.

### The relative expression and abundance of the outer membrane proteins differ between treatments and lifestyles of *V*. *cholerae*

**Fig 6:**
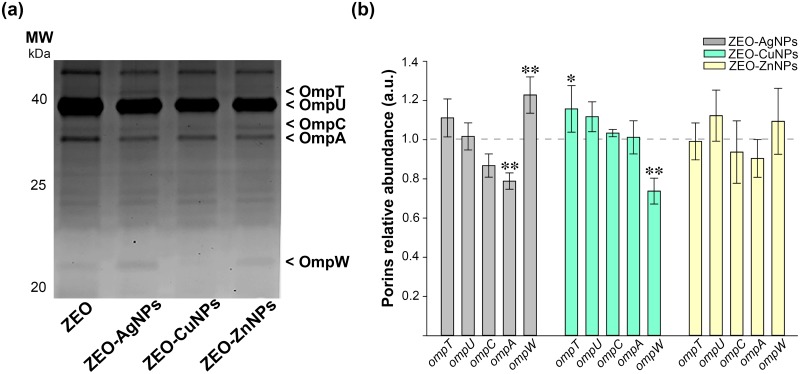
Relative abundance of the outer membrane proteins in pellicle biofilms (PB) after nanocomposites treatment. (a) SDS-PAGE of OMPs fractionation samples of PB exposed to ZEO (control), ZEO-AgNPs, ZEO-CuNPs and ZEO-ZnNPs. The OMPs are indicated by arrowheads. (b) OMPs relative abundance of PB treated at 1 μg/ml of ZEO-AgNP (gray), 420 μg/ml of ZEO-CuNPs (green) and ZEO-ZnNPs (yellow). The abundance of each OMP with ZEO treatment was considered as 1 (horizontal line). Densitometric analysis of SDS-PAGE gels was performed with ImageJ software from 3 independent OMPs purification experiments (n = 3). Significant changes are marked with asterisk: * p-value < 0.05, ** p-value <0.01.

**Fig 7:**
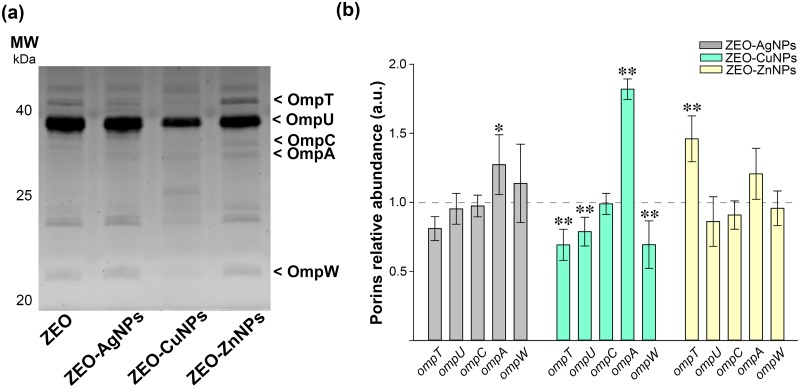
Relative abundance of the outer membrane proteins in surface-attached biofilms (SB) after nanocomposites treatment. (a) SDS-PAGE of OMPs fractionation samples of SB exposed to ZEO (control), ZEO-AgNPs, ZEO-CuNPs and ZEO-ZnNPs. The OMPs are indicated by arrowheads. (b) OMPs relative abundance of SB treated at 1 μg/ml of ZEO-AgNP (gray), 420 μg/ml of ZEO-CuNPs (green) and ZEO-ZnNPs (yellow). The abundance of each OMP with ZEO treatment was considered as 1 (horizontal line). Densitometric analysis of SDS-PAGE gels was performed with ImageJ software from 3 independent OMPs purification experiments (n = 3). Significant changes are marked with asterisk: * p-value < 0.05, ** p-value <0.01.

Next, we explored the role of outer membrane proteins (OMPs) as bacterial detoxification systems to expel or avoid toxic effects of nanocomposites. In that sense, OMPs were isolated by fractionation from PB and SB treated with ZEO-AgNPs, ZEO-CuNPs, and ZEO-ZnNPs (Figs 6–7). The *V*. *cholerae* OMPs were identified by molecular weight, OmpT (41 kDa), OmpU (39 kDa), OmpC (35 kDa), OmpA (34 kDa), and OmpW (22 kDa), and also, by immunobloting for the OmpT and OmpU (S6 Fig). It is important to notice the differences in the OMPs profile between PB and SB exposed to ZEO (first line, Figs 6A and 7A, respectively), where the relative abundance of OmpT, OmpC and OmpA varies between PB and SB in our control treatment.

We then compared OMPs profile of *V*. *cholerae* PB and SB exposed to the nanocomposites. Densitometric analysis revealed changes in the relative abundance of OMPs. In PB, the ZEO-AgNPs treatment decreases the OmpA expression, whereas increases the OmpW expression (Fig 6B, grey bars). On the other hand, ZEO-CuNPs treatments increase the OmpT expression and decrease the OmpW expression significatively (Fig 6B, green bars). Interestingly, ZEO-ZnNPs treatment does not change the expression of any OMPs in PB (Fig 6B, yellow bars).

In SB, the ZEO-AgNPs treatment only increased the OmpA abundance (Fig 7B, gray bars). In contrast, the ZEO-CuNPs treatment reduced the relative abundance of OmpT, OmpU and OmpW, and at the same time, the OmpA abundance was increased (Fig 7B, green bars). SB exposed to ZEO-ZnNPs showed an increment on relative abundance of the OmpT (Fig 7B, yellow bars). In summary, OMPs abundance seems to be specific for both forms of *V*. *cholera*e lifestyles–PB and SB-, and was clearly modified by nanocomposite treatments. It is important to note that the relative abundance of OmpC did not change between treatments or both lifestyles, whereas the OmpW levels were consistently reduced in ZEO-CuNPs treatments in both, PB and SB.

## Discussion

Biofilms tolerance has become a serious clinical concern due to their formidable resistance to conventional antibiotics and prevalent virulence. For this reason, there is a need to develop alternative antimicrobial agents to eradicate biofilms and prevent their formation. Metallic nanoparticles have been recently found to be a promising cure for microbial infection with a low chance of developing bacterial resistance.

In this study, the antimicrobial effect of Ag, Cu, and Zn NPs embedded into a zeolite matrix were tested in three different *V*. *cholerae* lifestyles: pellicle biofilm (PB), planktonic cells, and surface-attached biofilm (SB), to assess the sensitivity to metallic NPs depending on the cellular arrangements. MIC and MBEC assays showed that PB are the more sensitive to the NPs by preventing its formation at low ZEO-AgNPs concentrations, and even if cells remain viable in treatments with ZEO-CuNPs and ZEO-ZnNPs, a significant reduction in CFU/ml could be detected (Fig 1A and 1C). Interestingly, planktonic cells are more resistant to the nanocomposites tested, however, it is important to note that ZEO-NPs are sunk at the bottom of the well and that bacteria-nanocomposite direct contact might be a reason for having a higher antimicrobial efficiency on SB. Inhibition of biofilm formation by blocking the bacterial attachment to surfaces might be an alternative method to fight with biofilm-associated infections. Although there is a lower bacterial population, SEM images from PB and SB revealed no changes in bacteria morphology in the three treatments analyzed (Figs 2 and 4).

It is important to mention the low toxicity found for ZEO-CuNPs and ZEO-ZnNPs, whose antimicrobial effects as zero-dimensional metallic nanoparticles are well documented [34,35]. The oxidation state of the CuNPs and ZnNPs within the zeolite matrix seems to be fundamental for its antimicrobial activity. In that sense, XPS analysis showed the NPs for all nanocomposites can be considered with a core-shell type structure where the metallic cores of Ag, Cu, or Zn are covered by oxide species (S3 Fig).

At transcriptional level (Fig 5A and 5B), the biofilm and matrix-related genes expression showed a significant increase in *vpsL* expression during ZEO-CuNPs treatment in PB. The importance of *vpsL* in PB has been reported [36], showing that *vpsL* is essential for PB and SB formation as well as VPS production. Surprisingly, *vpsL*, *rbmA* and *bap1* expression decreased in SB during ZEO-ZnNPs treatment and, in agreement with the reduction of CFU/ml in PB and SB during this treatment, this result suggests a weaker *V*. *cholerae* SB. It is important to notice that gene expression was analyzed after 48 hours of incubation in order to obtain cell aggregates, which could be a time point where cells might be already adapted to the NPs and mature PB and SB formed. For the same reason, changes in expression of the master positive regulator of biofilm formation VpsR might not be detected. Early time points will be necessary to analyze in order to understand the adaptation mechanism to NPs by *V*. *cholerae*. Additionally, the effect of ZEO-NPs on already formed PB and SB is an important subject to address anti-biofilms strategies.

Due to the mechanism of action of NPs against other Gram-negative bacteria, specifically targeting the membrane integrity [37], we evaluated changes in OMPs expression under the different NPs treatments (Figs 6 and 7). OMPs fractionation allowed us to identify the most abundant pore-forming proteins (porins) in *V*. *cholerae*: OmpT and OmpU, and the OMPs: OmpC, OmpA and OmpW. Interestingly, ZEO-CuNPs downregulate three of the OMPs OmpT, OmpU, and OmpW in SB, while ZEO-ZnNPs only affects the expression of the OmpT in SB. It is important to notice that OmpC, does not change its expression with any treatment and in any condition. It is already known that bacterial OMPs play a role in the response to environmental changes, as well in the transport of iron, phosphates, sugars, hydrophilic and low molecular weight molecules, particularly, the selective exclusion of anionic detergents [38,39]. OmpT and OmpU porins also play an important role in the antibiotics flow into the cell [40]. Moreover, a role of OmpU on bile resistant and biofilm formation was reported on *V*. *angullarum* [41]. In *V*. *cholerae*, the resistance to bile has been associated with the OmpU small pore size [39]. The co-regulation of virulence factors -including OMPs- has been shown to alter biofilm formation, so it is important to determine the association between OMPs and biofilm against metallic NPs in order to employ them as a broad-spectrum biofilm-inhibition agent for designing new strategies to eradicate biofilms and cholera disease.

In summary, the antimicrobial activity of Ag, Cu and Zn nanocomposites were evaluated on *V*. *cholerae* PB, planktonic cells, and SB. Although the data obtained are not sufficient to describe the *V*. *cholerae* response mechanism against antimicrobial nanocomposites, changes on gene expression and protein abundance of those genes and proteins involved with biofilm formation and detoxification mechanisms were observed and a clear difference were observed between the two biofilm lifestyles tested (PB and SB). These results allow us to begin understanding *V*. *cholerae* resistance mechanisms against antimicrobial nanocomposites at different levels. However, a more deeply analysis of the transcriptional and proteomic changes during NPs treatment will be needed to understand differences in the molecular mechanism between PB and SB in *V*. *cholerae* and other pathogenic bacteria.

## Supporting information

S1 Fig Nanocomposites characterization by scanning electron microscopy (SEM). Images of (a) ZEO (matrix), and nanocomposites (b) ZEO-AgNPs, (c) ZEO-CuNPs, and (d) ZEO-ZnNPs. Representative images are shown.
(TIF)

S2 Fig Nanocomposites characterization by transmission electron microscopy (TEM) before and after 48h of culture media treatment. Images of the nanocomposites (a) ZEO-AgNPs, (b) ZEO-CuNPs, and (c) ZEO-ZnNPs. Representative images are shown.
(TIF)

S3 Fig Nanocomposites characterization by high resolution XPS spectra of the nanocomposites. (a) ZEO-AgNPs, (b) ZEO-CuNPs, and (c) ZEO-ZnNPs.
(TIF)

S4 Fig Structural visualization of non-treated pellicle biofilm (PB), planktonic cells and surface-attached biofilm (SB) by scanning electron microscopy (SEM). *V*. *cholerae* cultured without any nanocomposite (control), and ZEO treatment (zeolite matrix without metallic nanoparticles). Representative images of n = 3 biological replicates.
(TIF)

S5 Fig Structural visualization of *V*. *cholerae* planktonic cells over-time by scanning electron microscopy (SEM). *V*. *cholerae* cultured without nanocomposites (control), or ZEO (zeolite matrix without metallic nanoparticles). Representative images of n = 3 biological replicates.
(TIF)

S6 Fig Relative abundance of the outer membrane porins OmpT and OmpU by immunoblotting. OmpT (41 kDa), OmpU (39 kDa). Representative images are shown.
(TIF)

S1 Table Zeta potential (mV) of the nanocomposites before and after 48h of culture media treatment. (XLSX)
