# Supplementary figures and images for: Effect of antimicrobial nanocomposites on Vibrio cholerae lifestyles: Pellicle biofilm, planktonic and surface-attached biofilm
Source: PLoS One. 2019 Jun 12;14(6):e0217869. doi: 10.1371/journal.pone.0217869 (PMC6561565; doi:10.1371/journal.pone.0217869)

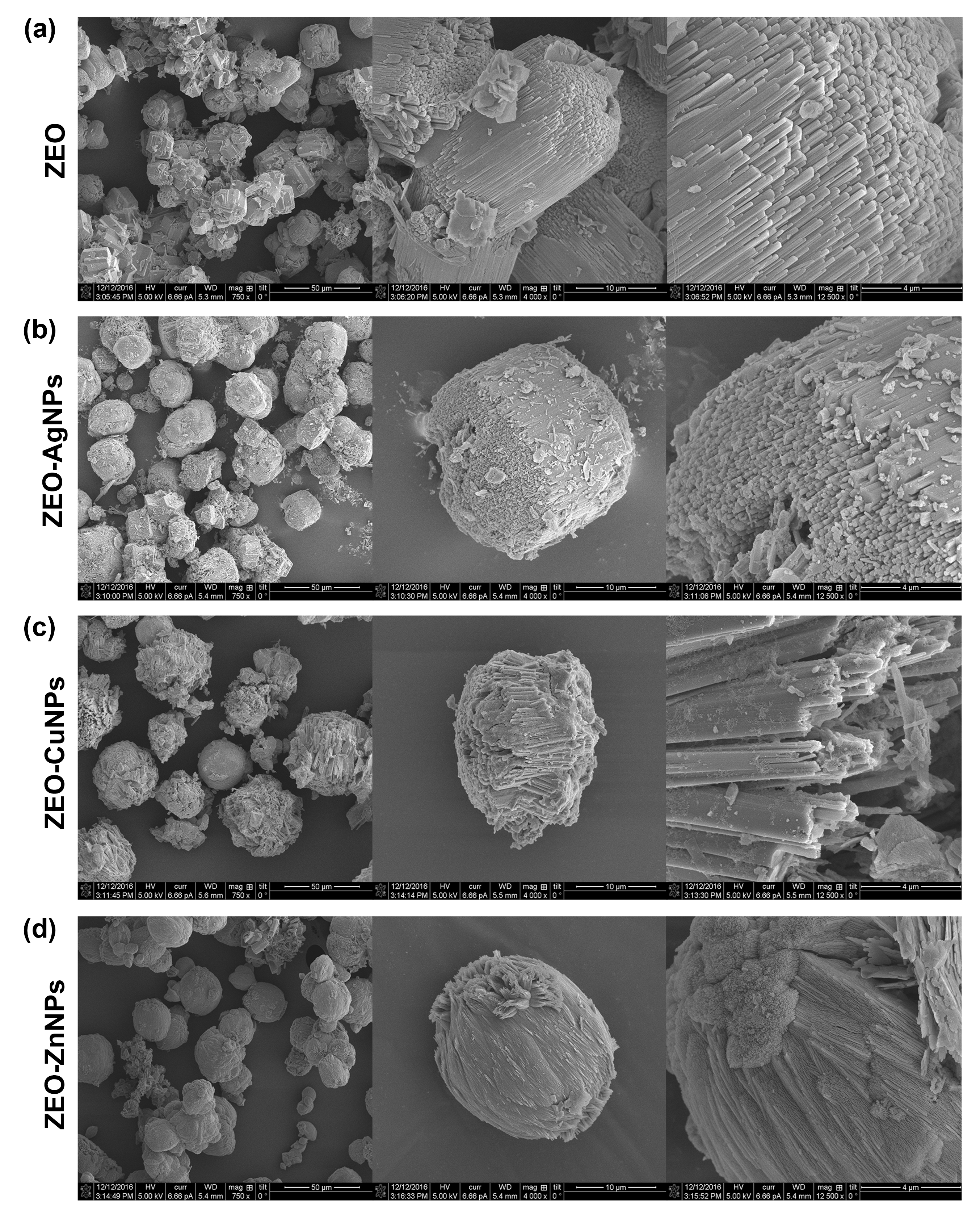

Supplement: S1 Fig — Images of (a) ZEO (matrix), and nanocomposites (b) ZEO-AgNPs, (c) ZEO-CuNPs, and (d) ZEO-ZnNPs. Representative images are shown. (TIF) [file pone.0217869.s001.tif]

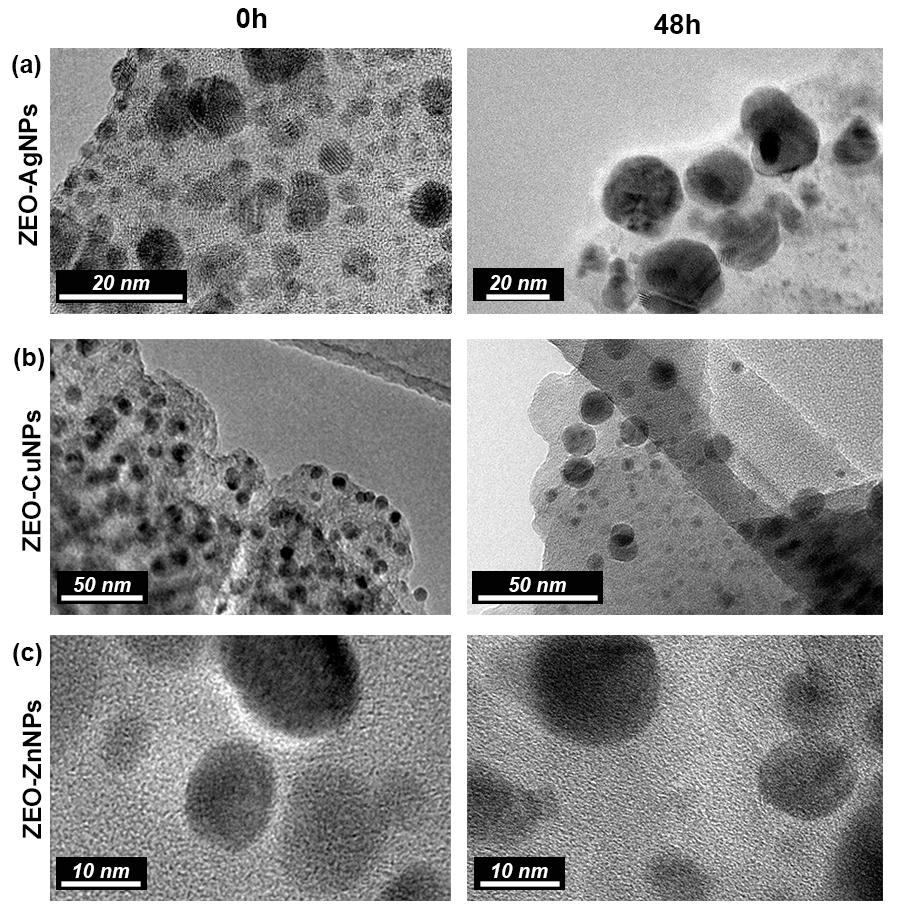

Supplement: S2 Fig — Images of the nanocomposites (a) ZEO-AgNPs, (b) ZEO-CuNPs, and (c) ZEO-ZnNPs. Representative images are shown. (TIF) [file pone.0217869.s002.tif]

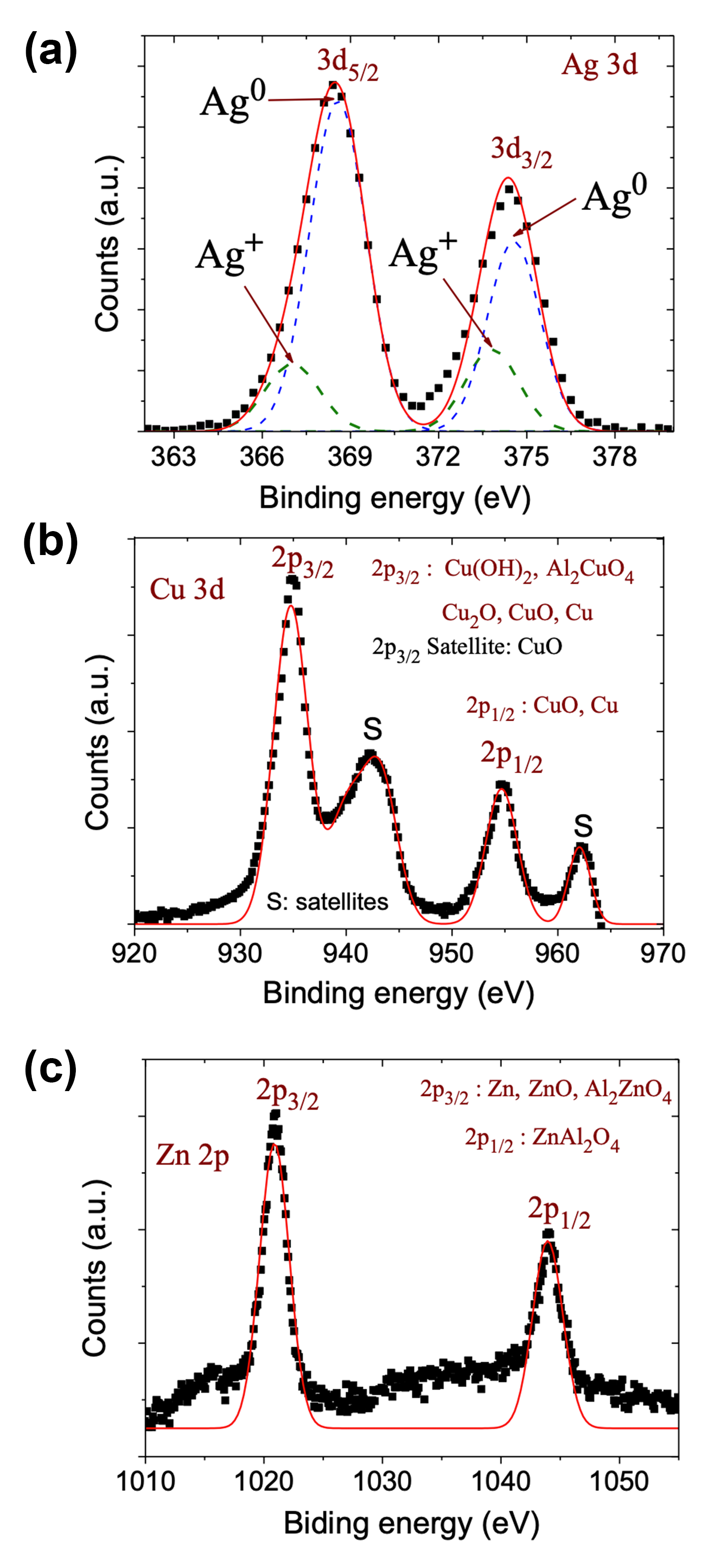

Supplement: S3 Fig — (a) ZEO-AgNPs, (b) ZEO-CuNPs, and (c) ZEO-ZnNPs. (TIF) [file pone.0217869.s003.tif]

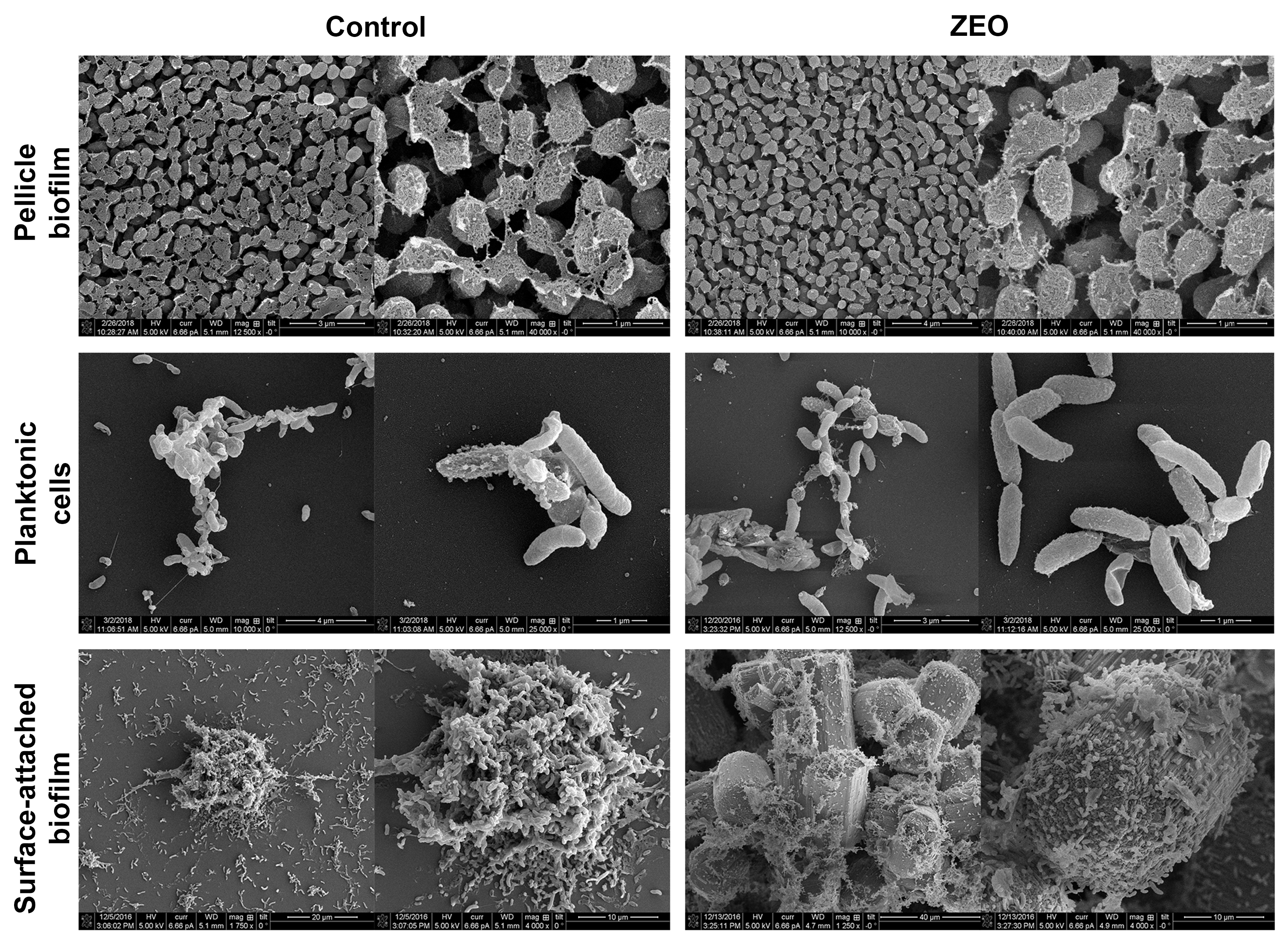

Supplement: S4 Fig — V. cholerae cultured without any nanocomposite (control), and ZEO treatment (zeolite matrix without metallic nanoparticles). Representative images of n = 3 biological replicates. (TIF) [file pone.0217869.s004.tif]

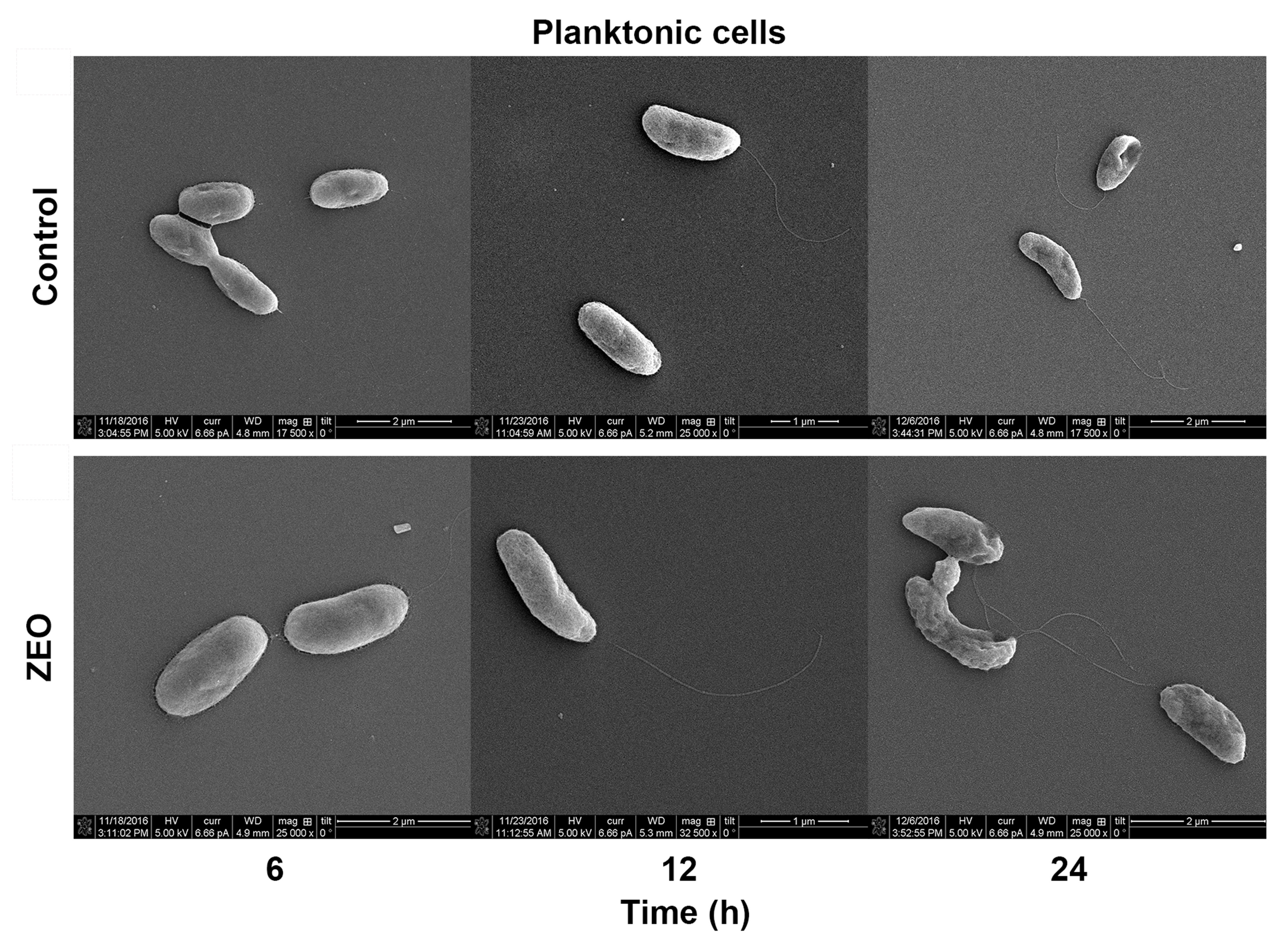

Supplement: S5 Fig — V. cholerae cultured without nanocomposites (control), or ZEO (zeolite matrix without metallic nanoparticles). Representative images of n = 3 biological replicates. (TIF) [file pone.0217869.s005.tif]

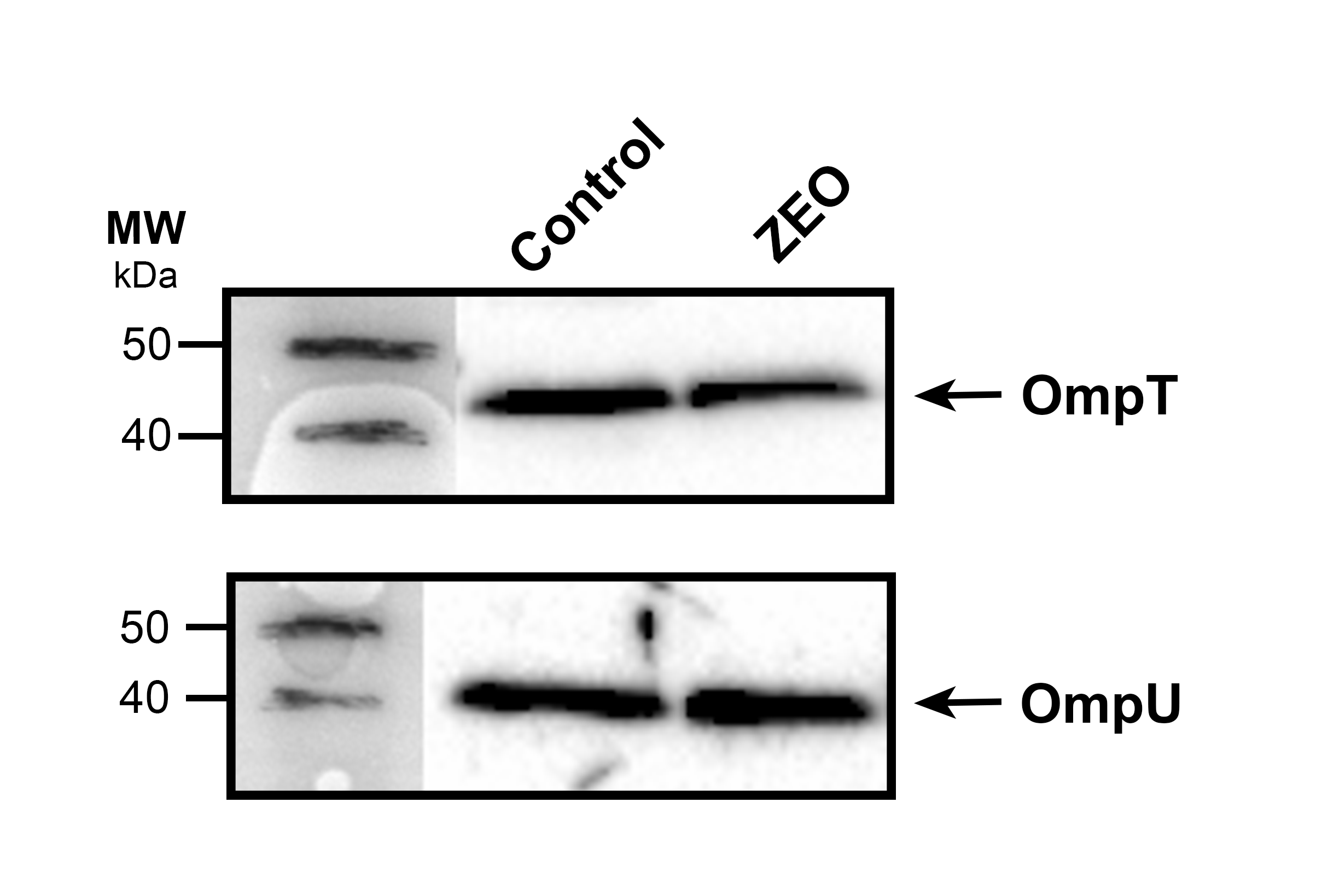

Supplement: S6 Fig — OmpT (41 kDa), OmpU (39 kDa). Representative images are shown. (TIF) [file pone.0217869.s006.tif]
